# Plasmapheresis as an Ischaemic Stroke Treatment: A Rare Case of Acquired Thrombotic Thrombocytopenic Purpura

**DOI:** 10.7759/cureus.72762

**Published:** 2024-10-31

**Authors:** Francisco Barreto, Sofia Nóbrega, Rui Fernandes, Carolina I Carvalhinha, Teresa Faria

**Affiliations:** 1 Internal Medicine, Hospital Central do Funchal, Funchal, PRT

**Keywords:** acquired thrombotic thrombocytopenic purpura, adamts13 activity, plasmapheresis, stroke, thrombotic microangiopathy

## Abstract

Acquired thrombotic thrombocytopenic purpura (acquired TTP) is a rare clinical syndrome caused by a decreased ADAMST13 activity, leading to systemic microvascular thrombotic events, with high mortality rates when the diagnosis and treatment are delayed. The authors report an acquired TTP in a patient with cerebrovascular disease. The aim is to emphasize the importance of considering atypical acquired TTP clinical presentations in order to optimize diagnostic and treatment approaches, minimize possible sequels, and improve the prognosis.

## Introduction

Acquired thrombotic thrombocytopenic purpura (acquired TTP) is thrombotic microangiopathy (TMA) that occurs with thrombocytopenia, microangiopathic hemolytic anemia, and microvascular occlusion [1]. It has a global annual incidence of two to six cases per million people and occurs more frequently in women, with a median age of 40 years [2]. This TTP results from an acquired decrease of the von Willebrand factor-cleaving protease ADAMTS13 due to inhibitory autoantibodies, causing uncontrolled microthrombi formation, which can lead to end-organ ischemia and damage [3]. There is a considerable clinical overlap with other thrombotic microangiopathies, such as hemolytic uremic syndrome (HUS), thus the diagnostic challenge [4]. Acquired TTP is considered a medical emergency with high mortality rates, whether its diagnostic and therapeutic approaches are delayed. Moreover, this clinical syndrome is characterized by a crucial recurrence risk [5]. The authors present a case report of an atypical presentation form of acquired TTP and its respective treatment and clinical evolution.

## Case presentation

A 75-year-old woman from Madeira Island in Portugal, with past history of atrioventricular block with a pacemaker, paroxistic atrial fibrillation under edoxaban treatment, obesity, essential hypertension and Sjögren syndrome, is admitted to the emergency room (ER) with a recent transient episode of dysarthria, right arm paresis, labial commissure deviation to the left side, and dysphagia for liquids, witnessed by her daughter. The patient also mentioned a right hemicranial headache. Two days before, the patient had developed occipital headaches and dysarthria and reported having undergone a cranial-encephalic computed tomography at the ER, which only showed chronic sinusopathy without other major changes. Upon objective examination in the emergency department (ER), the patient was conscious, cooperative, and oriented allo- and autopsychically, with fever (tympanic temperature of 38.7°C), hypertensive blood pressure profile, normal heart rate, and without any abnormality in cardiopulmonary and abdominal examination. Neurological examination showed no dysarthria or aphasia, and a slight deviation of the labial commissure to the right was notable, without other relevant findings in the remaining clinical examination. The patient remained under clinical surveillance at the ER, having her condition worsened again with aphasia and right hemiparesis a few hours after admission. Table 1 shows the blood test results where the patient presented anemia with hemoglobin of 10.4 g/dL, thrombocytopenia with 26,000 platelets/μL, mild renal dysfunction, urea of 57.4 mg/dL, creatinine of 1.02 mg/dL, glucose of 204 mg/dL, lactate dehydrogenase (LDH) of 662 U/L, and elevated DD-dimers (7,227 ng/mL). The peripheral blood smear findings were compatible with TMA, namely, thrombocytopenia, keratocytes, schistocytes, and microspherocytes. The direct and indirect Coombs tests were negative. Lumbar puncture results showed less than two cells with no predominant lineage.

**Table 1:** Preclinical results - hemogram, chemistry, coagulation tests, and immunology dL - deciliter; g - gram; L - liter; mg - milligram; µL - microliter; PT - prothrombin time; aPTT - activated partial thromboplastin time; dL - deciliter; mg - milligram; mL - milliliter; ng - nanogram; s - seconds

Hemogram	Results	Normal range
Leukocytes/mm^3^	9100	3.500-10.000
Hemoglobin (g/dL)	10.4	12.0-16.0
Platelets/µL	26000	150.000-400.000
Peripheral blood smear	Thrombocytopenia confirmed, keratocytes, schistocytes and microspherocytes	-
Chemistry	Results	Normal range
Urea (mg/dL)	57.4	5-20
Creatinine (mg/dL)	1.02	0.7-1.3
Lactate dehydrogenase (units/L)	662	120-246
Total Bilirrubin (mg/dL)	1.1	0.2-1.2
Haptoglobin (mg/dL)	< 7.69	40-165
Coagulation tests	Results	Normal range
D-dimer (ng/mL)	7227	< 500
PT(s)/INR/aPTT (s)	12.4/1.06/20.1	11-14/0.8-1/21-35
Fibrinogen (mg/dL)	461	200-400
Immunology	Results	-
Antineutrophil cytoplasmatic antibodies (ANCAs)	Negative	-
Antinuclear antibodies (ANAs)	Positive	-
Anti-SSA and Anti-SSB antibodies	Positive	-
C3 and C4	Normal	-
Direct and indirect Coombs test	Negative	-

**Figure 1:**
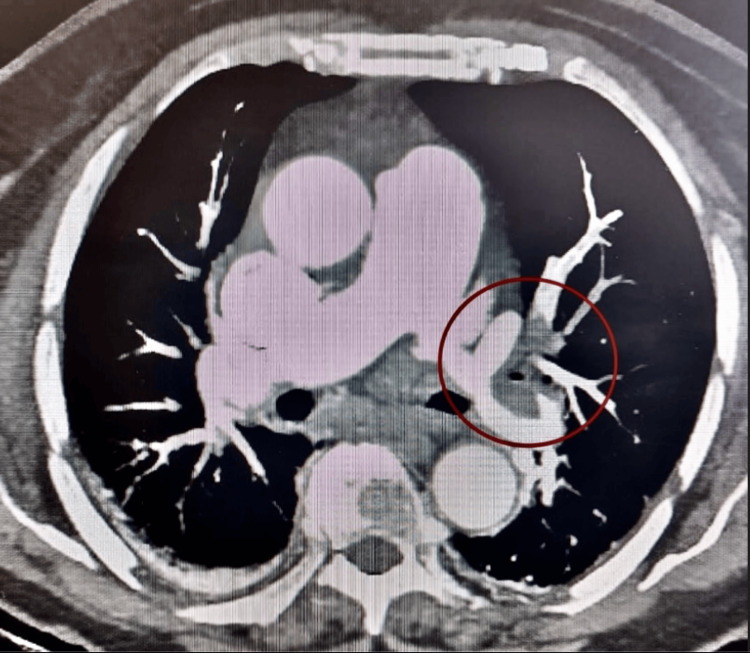
Pulmonary embolism The red circle shows the left lobar branch thrombus.

Cranial computed tomography angiography (CTA) did not reveal recent ischemic lesions (ASPECTS 10) nor intracranial blood densities. Due to the new onset dyspnea and desaturation, a thoracic CTA was performed, showing filling defects in lobar branches compatible with acute pulmonary thromboembolism (PTE). Figure 1 shows the proximal left lobar branch thrombus in the thoracic CTA.

Due to the anemia with schistocytes in the morphological study of peripheral blood, thrombocytopenia, fever, new and fluctuating neurological dysfunction, and thrombotic phenomena, acquired thrombotic thrombocytopenic purpura was assumed to be the most likely diagnosis. Plasma ADAMTS13 activity was not tested as it was unavailable in our laboratory. The patient started corticosteroid therapy (methylprednisolone) and plasmapheresis with plasma exchange (PTE), and platelet counts recovered to >150,000/mm^3^ and LDH to normal values. However, upon discontinuation of therapy, the patient relapsed with hemolysis and an abrupt drop in platelet count but no neurological symptoms, imposing the need to reinstitute daily plasma exchanges associated with weekly administration of rituximab (1 g intravenously - dose of 375 mg/m^2^). There was subsequent normalization of hematological values, a drop in LDH, and no signs of hemolysis in the peripheral blood smear. In a multidisciplinary meeting with internal medicine, transfusion medicine, and hematology, it was decided to stop PTE and begin weaning from corticosteroid therapy. The patient was discharged with clinical stability without any neurological deficits and normal blood counts, under enoxaparin.

## Discussion

TTPa is a rare nosological entity hallmarked by TMA and dysfunctional ADAMTS13 enzymatic activity, with a challenging diagnosis considering its acute nature with signs and symptoms common to other thrombotic microangiopathies and high mortality [5]. Therefore, it requires a swift therapeutic approach in order to minimize risks and long-term sequelae.

The diagnostic criteria are essentially the presence of a classic pentad of TTP findings, such as thrombocytopenia, microangiopathic hemolytic anemia, neurological abnormalities, fever, and renal dysfunction. There is also increased interest in using the PLASMIC score in patients with suspected TTP who might benefit from early initiation of plasma exchange while awaiting ADAMTS13 results [6]. This score's main determinants are platelets < 30,000/uL, presence of hemolysis (reticulocytes >2.5%, total bilirubin >2.0 or decreased haptoglobin), absence of active cancer, absence of stem cell or organ transplant, mean corpuscular volume < 90 fL, INR of <1.5, and Cr of < 2.0 mg/dL [7].

In the case described, the patient presented a clinical condition suggestive of a cerebrovascular event. However, there were some atypical clinical and laboratory findings that are not usually consistent with a transient ischemic attack (TIA) or minor stroke, namely, fever, new mild renal dysfunction, AHMA, and thrombocytopenia. Additionally, and given the past history of autoimmune disease (Sjögren syndrome) and the wavering neurological deficits, acquired TTP was considered the main clinical hunch.

According to the state of the art, PTE should be immediately initiated until the conditions are stabilized [8]. Relapsed patients are treated with daily plasma exchange, steroids, and rituximab [8]. In this case, the patient was empirically started on methylprednisolone and PTE, with substantial improvement over the subsequent five days, emphasizing the importance of early therapeutic initiation in reducing morbidity and mortality.

The refractory nature of this disease is also noteworthy, demonstrated in this case by the immediate relapse upon discontinuation of treatment, with the need to initiate immunosuppressive therapy with rituximab associated with corticosteroid therapy and plasma exchange, with a favorable evolution of the condition.

## Conclusions

Acquired TTP is a rare syndrome with a challenging diagnosis taking into account the unspecific signs and symptoms common to other thrombotic microangiopathies, its acute nature, and the highly associated mortality, which in turn requires a rapid therapeutic approach in order to minimize death risk and long-term sequelae.

The present case highlights the importance of considering less recognized etiologies such as acquired TTP in the differential diagnosis of TIA/minor stroke.
